# Sensitivity of effective dose to changes in tissue weighting factors

**DOI:** 10.1007/s00411-025-01143-1

**Published:** 2025-08-26

**Authors:** Thomas Otto

**Affiliations:** CERN, European Organization for Nuclear Research, 1211 Genève 23, Switzerland

**Keywords:** Effective dose, Tissue weighting factor, Operational quantities

## Abstract

Effective dose *E* is the radiological protection quantity for stochastic health effects from whole-body exposure to ionising radiation. It is defined as a weighted sum of average absorbed doses in 15 selected tissues (organs), with weighting factors for radiation effectiveness and tissue sensitivity. This paper analyses the sensitivity of effective dose *E* with respect to the tissue weighting factors $w_{\textrm{T}}$. It shows that a change of any single weighting factor by 100% results in relative changes of effective dose by a few percent for photons above an energy of 50 keV and for neutrons. Larger variations are observed for neutrons with an energy around 1 MeV and photons with low energies. The organs close to the body surface, breast and skin, are identified as the cause for these larger variations. Absolute changes of effective dose remain small, with exception for persons predominately exposed to low-energy photon radiation.

**Supplementary Information:**

The online version contains supplementary material available at 10.1007/s00411-025-01143-1.

## Introduction

In radiological protection, effective dose *E* is the protection quantity for stochastic radiation detriment. Dose limits for whole-body exposure are expressed in terms of effective dose. *E* has been defined by the International Commission on Radiological Protection, ICRP, in its Publication 60 (1991) and revised in ICRP Publication 103 (2007). In its present form it can be expressed as:

1 $$\begin{aligned} & H_{\textrm{T}} = \sum _R{w_{\textrm{R}} D_{\textrm{T,R}}}, \end{aligned}$$

2 $$\begin{aligned} & E = \sum _T{w_{\textrm{T}} H_{\textrm{T}}}. \end{aligned}$$

In these equations, $D_{\textrm{T,R}}$ is the absorbed dose from radiation type *R* averaged over tissue (organ) *T* and $H_{\textrm{T}}$ the organ equivalent dose. In the second sum, the index *T* ranges from 1 to 15. It indicates 14 unique organs and tissues, and the remainder, grouping 14 tissues distributed over the body having a lower sensitivity to radiation. $w_{\textrm{R}}$ and $w_{\textrm{T}}$ are age- and sex averaged radiation- and tissue-weighting factors, taking into account the relative effectiveness of radiation types and the radiation sensitivity of organs. The tissue weighting factors $w_{\textrm{T}}$ have values between 0.01 and 0.12, with $\sum {w_{\textrm{T}}} = 1$.

For a given radiation field, effective dose is evaluated for a reference person: Eq. 1 is evaluated by Monte-Carlo radiation transport calculations separately for male and female anthropomorphic reference voxel phantoms, described in (ICRP 2009). The sex-specific organ equivalent doses are then averaged to obtain *E*:

3 $$\begin{aligned} E = \sum _T{ w_{\textrm{T}} \left( \frac{H_{\textrm{T}}^{\textrm{M}} + H_{\textrm{T}}^{\textrm{F}}}{2}\right) } \end{aligned}$$

**Fig. 1:**
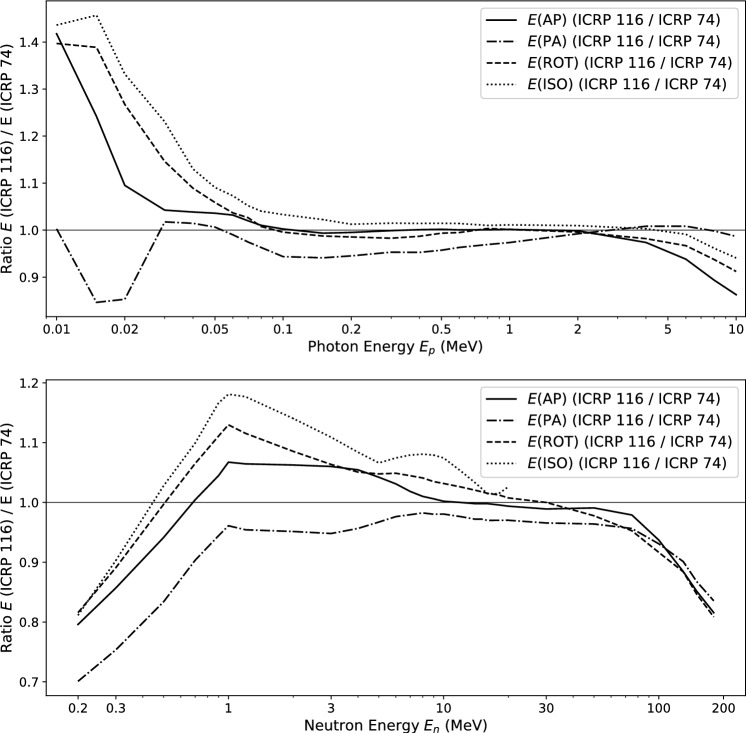
Comparison between the fluence-to-effective dose conversion coefficients from ICRP Publication 116 (following definitions in ICRP Publication 103) to those from ICRP Publication 74 (following definitions in ICRP Publication 60), for four different orientations of the radiation field. For neutrons, the radiation weighting factor $w_{\textrm{R}}$ for energies below a few 100 keV was essentially halved, therefore the comparison is shown for energies larger than 200 keV

Between ICRP Publications 60 and 103, new biological and physical information led to a revision of the radiation and tissue weighting factors (Table 1), and anatomically realistic voxel phantoms (ICRP 2009) were introduced for the calculation. Conversion coefficients to effective dose for broad beams, covering the whole body and coming from standard directions of incidence (AP, PA, ISO, ...) following the previous definition of *E* in ICRP Publication 60 were published in ICRU Report 57 and in ICRP Publication 74 (ICRU 1999; ICRP 1996) and for the present definition of *E* in ICRP Publication 116 (2010). The change of the definition of *E* led to changes in the energy-dependent conversion coefficients from fluence to effective dose (Fig. 1).

**Table 1:** Tissue weighting factors $w_{\textrm{T}}$ in ICRP Publications 60 (1991) and 103 (2007)

Tissue (Organ)	ICRP 60	ICRP 103
Bone marrow, colon, lungs, stomach	0.12	0.12
Breast, remainder^1^	0.05	0.12
Gonads	0.20	0.08
Thyroid, oesophagus, liver, bladder	0.05	0.04
Bone surface, skin	0.01	0.01
Brain	–^2^	0.01
Salivary glands^3^	–	0.01

^1^In ICRP publication 103, the remainder groups the following tissues: adrenals, extrathoracic (ET) region, gall bladder, heart, kidneys, lymphatic nodes, muscle, oral mucosa, pancreas, prostate (for male), small intestine, spleen, thymus and uterus/cervix (for female)

^2^Included in the remainder

^3^Tissue added in ICRP Publication 103

The Commission states in ICRP Publication 116 (2010) that conversion coefficients to effective dose for photons with energies between 100 keV and 6 MeV show differences of a few % between ICRP Publications 74 and 116. At low photon energies, the differences are more pronounced, due to the differences in the contributions of organs close to the body surface, such as skin, breast and thyroid. For neutrons there is good agreement between the values of effective dose following definitions of Publication 60 or 103 for energies between 400 keV and 50 MeV. Outside of this interval, the influence of the modified radiation weighting factor $w_{\textrm{R}}$ for neutrons in ICRP Publication 103 is responsible for larger deviations.

In practice, effective dose cannot be measured because it is not a point-like quantity but defined over the extended volume of the human body. The International Commission on Radiation Units and Measurements, ICRU, introduced operational quantities for external radiation, personal dose equivalent $H_{\textrm{p}}$(10) and ambient dose equivalent $H^*$(10) (ICRU 1993). These point-like quantities serve for the design, type-testing and calibration of radiation protection dosimeters and monitoring instruments and are supposed to give conservative estimates of effective dose. Introduced in 1993, the operational quantities did not change with the revision of effective dose in 2007 (ICRP 2007). The indication of radiation protection dosimeters, survey instruments and monitors remained unchanged and the revision of effective dose went largely unnoticed by radiation protection practitioners.

It is possible that the forthcoming general recommendations of the ICRP, expected for the beginning of the 2030s, will introduce further modifications of the weighting factors. Furthermore, improved anthropomorphic reference phantoms based on a tetrahedral mesh-representation have been introduced in ICRP Publication 145 (2020). This time, a change of the values of effective dose may have a more direct impact on practical radiation protection. The ICRU recommends in Report 95 (2020) new operational quantities, personal dose $H_{\textrm{p}}$ and ambient dose $H^*$. They are closely related to effective dose *E* because their conversion factors from fluence are calculated with the same phantoms and the same weighting factors as effective dose. This unified formalism follows the state of science and technology, it leads to a simplification of the system of radiation protection dosimetry and a more realistic representation of effective dose by the operational quantities. The two quantities are numerically equal where one would expect it, for example:

4 $$\begin{aligned} H^*(0^o)&= H_{\textrm{p}}(0^o) = E(\textrm{AP}), \nonumber \\ H^*(180^o)&= H_{\textrm{p}}(180^o) = E(\textrm{PA}) . \end{aligned}$$

The close relation of the proposed operational quantities in (ICRU 2020) to effective dose seems to imply a quasi-automatic change of the operational quantities (Gilvin et al. 2022). This would necessarily require the modification of the energy- and angle dependent response of personal dosimeters and radiation protection instruments. This automatism is contrary to the need for stability in quantities which serve to design and calibrate dosimeters and monitors, forming the metrological basis for practical radiation protection dosimetry.

This paper investigates with a simple model the potential magnitude of changes in the conversion coefficients for effective dose *E* provoked by changes of the tissue weighting factors $w_T$. It then discusses the significance of these changes for the operational quantities personal dose $H_p$ and ambient dose $H^*$ and possible strategies to cope with these.

## Method

To investigate the sensitivity of the numerical values of effective dose *E* to the tissue weighting factors $w_{\textrm{T}}$, a conservative model is introduced in which single weighting factors are changed by 100%. Effective dose-like quantities $E_{\mathrm {0,T'}}$ and $E_{\mathrm {2,T'}}$ are defined for each tissue T’. In the 15 quantities $E_{\mathrm {0,T'}}$ the weighting factor $w_{\mathrm {T'}}$ is set to zero, while its value is doubled in the quantities $E_{\mathrm {2,T'}}$ The other weighting factors are adjusted proportionally so that the relation $\sum {w_{\mathrm {T'}}}=1$ remains valid. Formally, this procedure can be expressed in the following way:

5 $$\begin{aligned} E_{\mathrm {0,T'}}&= \sum _T{w_{\textrm{T}}H_{\textrm{T}}} - w_{\mathrm {T'}}H_{\mathrm {T'}}\\& \quad \textrm{with} \quad \sum _T{w_{\textrm{T}}} - w_{\mathrm {T'}} = 1 \end{aligned}$$

6 $$\begin{aligned} E_{\mathrm {2,T'}}&= \sum _T{w_{\textrm{T}}H_{\textrm{T}}} + w_{\mathrm {T'}}H_{\mathrm {T'}}\\& \quad \textrm{with} \quad \sum _T{w_{\textrm{T}}} + w_{\mathrm {T'}} = 1 \end{aligned}$$

To simplify the expressions, it is assumed that here $H_{\textrm{T}}$ stands for the average of the male and female organ equivalent doses.

Table 2 demonstrates the weight factor modification on the example of the gonads.

**Table 2:** Changed tissue weighting factors $w_{\textrm{T}}$ for doubling or setting to zero the factor for the gonads $w_{\textrm{gonads}}$

Tissue (Organ)	ICRP 103	No gonads	Double gonads
Bone marrow, colon, lungs, stomach, breast, remainder	0.12	0.132	0.108
Gonads	0.08	0.0	0.16
Thyroid, oesophagus, liver, bladder	0.04	0.0441	0.0359
Bone surface, skin, brain, salivary glands	0.01	0.0110	0.00899

Numerical values are rounded to three significant digits

In the expressions Eqs. 5 and 6, the effect of increasing or decreasing the weighting factor of one tissue is compensated by the adjustment of the other factors so that the sum of the factors is one. Depending on the relative size of the organ equivalent doses $H_{\textrm{T}}$, either of the expressions $E_{\mathrm {0,T'}}$ and $E_{\mathrm {2,T'}}$ may become smaller or larger than the standard effective dose *E*. Due to the symmetry in the two expressions, the following relation is valid for all radiation field orientations and energies:

7 $$\begin{aligned} E - E_{\mathrm {0,T'}} = E_{\mathrm {2,T'}} - E . \end{aligned}$$

The described procedure leads for each direction of radiation incidence to a total of 30 effective dose-like quantities in which one tissue weighting factor has been significantly changed. For the evaluation of the expressions Eqs. 5, 6, the energy-dependent numerical values for $H_{\textrm{T}}^{\textrm{M}}$, $H_{\textrm{T}}^{\textrm{F}}$ are calculated according to Eq. 1 by multiplying the organ absorbed doses from the annex of ICRP Publication 116 (2010) with the radiation weighting factor $w_{\textrm{R}}$. This factor is unity for photons, and an energy-dependent function for neutrons. The evaluation covers the whole energy range for which conversion coefficients are published. The calculation yields two energy-dependent series of modified conversion coefficients, in the example from Table 2$E_{\mathrm {0,\,gonads}}$ and $E_{\mathrm {2,\,gonads}}$. Finally, for each tissue , the ratio between the modified values of effective doses and the canonical values of effective dose from (ICRP 2010) are calculated and plotted as a function of energy:

8 $$\begin{aligned} R_{\mathrm {0,\,T'}} = \frac{E_{\mathrm {0,\,T'}}}{E},\quad R_{\mathrm {2,\,T'}} = \frac{E_{\mathrm {2,\,T'}}}{E}. \end{aligned}$$

This procedure is repeated for all 15 tissues in the definition of *E*, for different orientations of the incident radiation field and for photons and neutrons.

While the extent of the potential modifications of tissue weighting factors in the future revision of the ICRP General Recommendations is unknown, this model, in which the radiation sensitivity of a tissue is either doubled or set to zero, should give a realistic impression about the order of magnitude of expected changes in effective dose *E*.

## Results

As described above, for each orientation of the radiation field, 30 different modified effective dose-like quantities $E_{\mathrm {0,T'}}$ and $E_{\mathrm {2,T'}}$ are calculated and put in relation with the standard values of effective dose. In this section, the results are given mainly in form of energy-dependent graphs of the ratios $R_{\mathrm {0,\,T'}}$ and $R_{\mathrm {2,\,T'}}$ for a selection of tissues and radiation field orientations. As a consequence of the relation Eq. 7 the two curves are symmetric to each other with respect to the line at unity.

### Photons

**Fig. 2:**
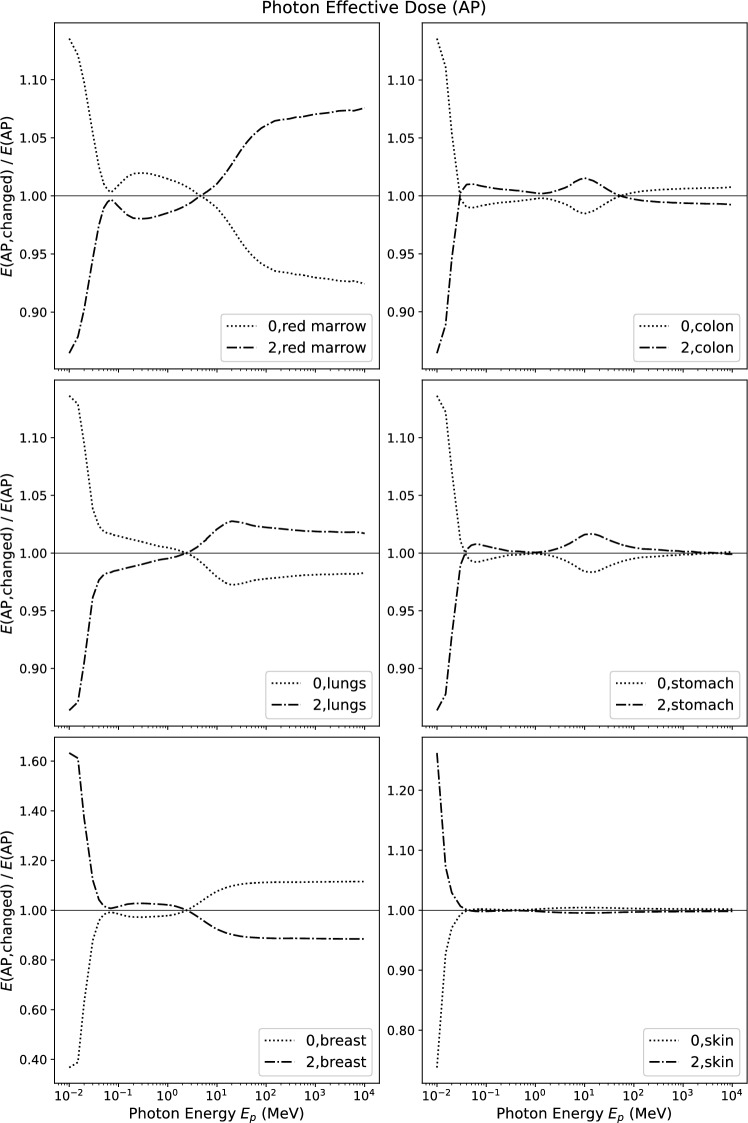
Relative change of effective dose *E* for photons in AP incidence when changing the tissue weighting factor indicated in the figure to zero or to its double

Figure 2 shows the relative change (Eq. 8) of effective dose in anterior-posterior irradiation geometry *E*(AP) for five of the tissues with weighting factor $w_{\textrm{T}}$ = 0.12 and for skin.

For photon energies above 50 keV, the relative changes are below 5% for most tissues, only modified weighting factors for red bone marrow and for the breast generate larger changes at high energies.

For low photon energies ($E_p < 50$ keV) the relative changes increase sharply for all modified tissue weighting factors. With decreasing photon energy, the depth of a tissue within the body becomes an increasingly important parameter, strongly influencing the value of the tissue dose conversion coefficient.

**Fig. 3:**
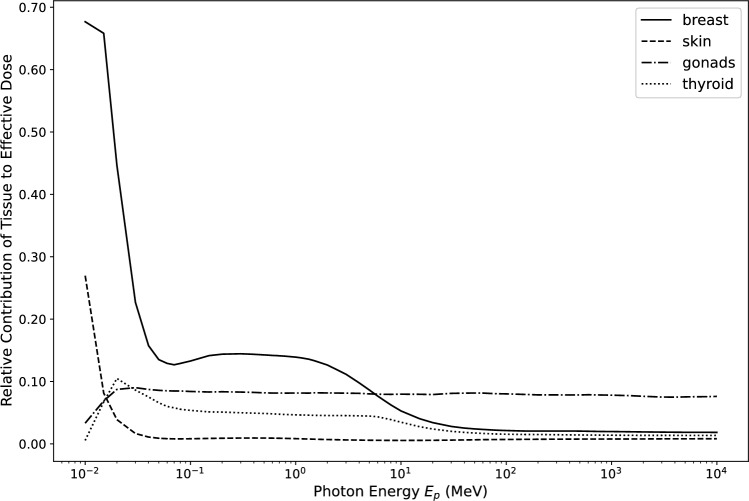
Contribution to effective dose *E*(AP) by the four tissues dominating at low energies. Shown is the expression $w_{\textrm{T}} \cdot H_{\textrm{T}}/E$

In AP irradiation geometry, the breast and the skin are the closest tissues to the body surface. At low energies, photons deposit a large part of their energy in them and their relative contribution to *E* exceeds all other contributions (Fig. 3). Thus, the most important deviations from *E* as published in (ICRP 2010) are observed for changed weighting factors of breast (60%) and skin (30%). When the weighting factors for breast or skin are doubled, the modified effective doses $E_{\textrm{2,T}}$(AP) increase significantly. When other tissue weighting factors are modified, a compensation effect is acting: at low photon energies, $E_{\textrm{2,T}}$(AP) is *larger* than *E*(AP) for breast and skin. For doubling any of the other tissues weighting factors $E_{\textrm{2,T}}$(AP) is *smaller* than *E*(AP), because the weighting factors for breast and skin *decrease* to compensate, and they alone dominate at low photon energies.

**Fig. 4:**
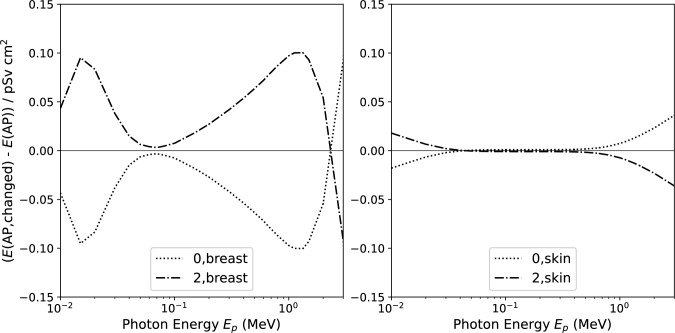
Difference between the modified and the standard effective dose *E* for photons in AP incidence when changing the tissue weighting factor for breast or skin to zero or to its double. The energy interval ranges from 10 keV to 3 MeV, including all relevant photon energies from X-ray and radionuclide emissions

The relative change of effective dose at low photon energies may give an erroneous impression of the absolute change of the quantity. The values of effective dose are very low at low photon energies, and the relative changes shown in Fig. 2 translate to modest changes of the conversion coefficient of less than 0.15 pSv $\text {cm}^2$ (Fig. 4).

**Fig. 5:**
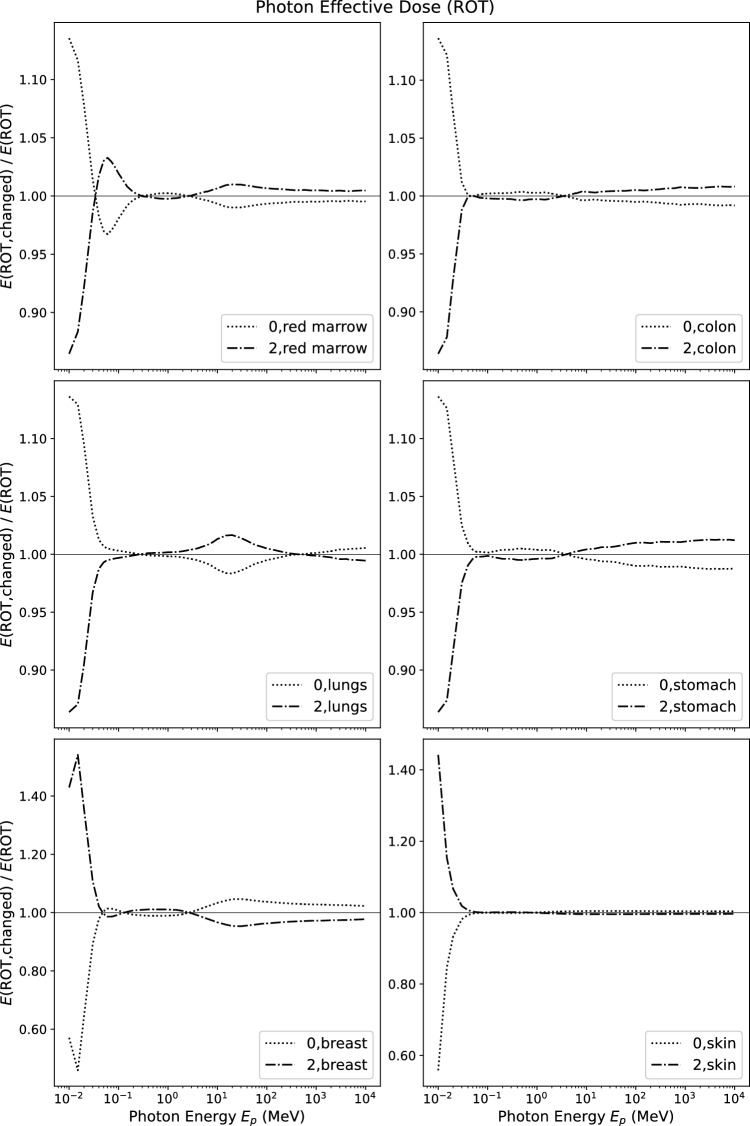
Relative change of effective dose *E* for photons in ROT incidence when changing the tissue weighting factor indicated in the figure to zero or to its double

Figure 5 shows the relative change (Eq. 8) of effective dose in rotational irradiation geometry *E*(ROT) for the same tissues as in Fig. 2. In rotational geometry, the radiation is equally incident from all directions orthogonal to the vertical symmetry axis of the anthropomorphic phantoms. This geometry is sometimes used as an approximation for workers moving in workplaces with many, distributed radiation sources. Here, the relative changes for effective dose at high photon energies are even smaller than for AP-orientation due to the averaging effect over all directions, which gives less importance to organs close to one body surface.

For low photon energies, the picture is similar to Fig. 2. The change in *E*(ROT) following a modification of $w_{\textrm{skin}}$ is even higher than in the case of AP-orientation, because independent of the direction of incidence the skin is the first tissue to be encountered by external radiation. The changes implied by a modification of $w_{\textrm{breast}}$ are smaller than for the AP-geometry because the breast is not impacted by irradiation from backward angles.

### Neutrons

**Fig. 6:**
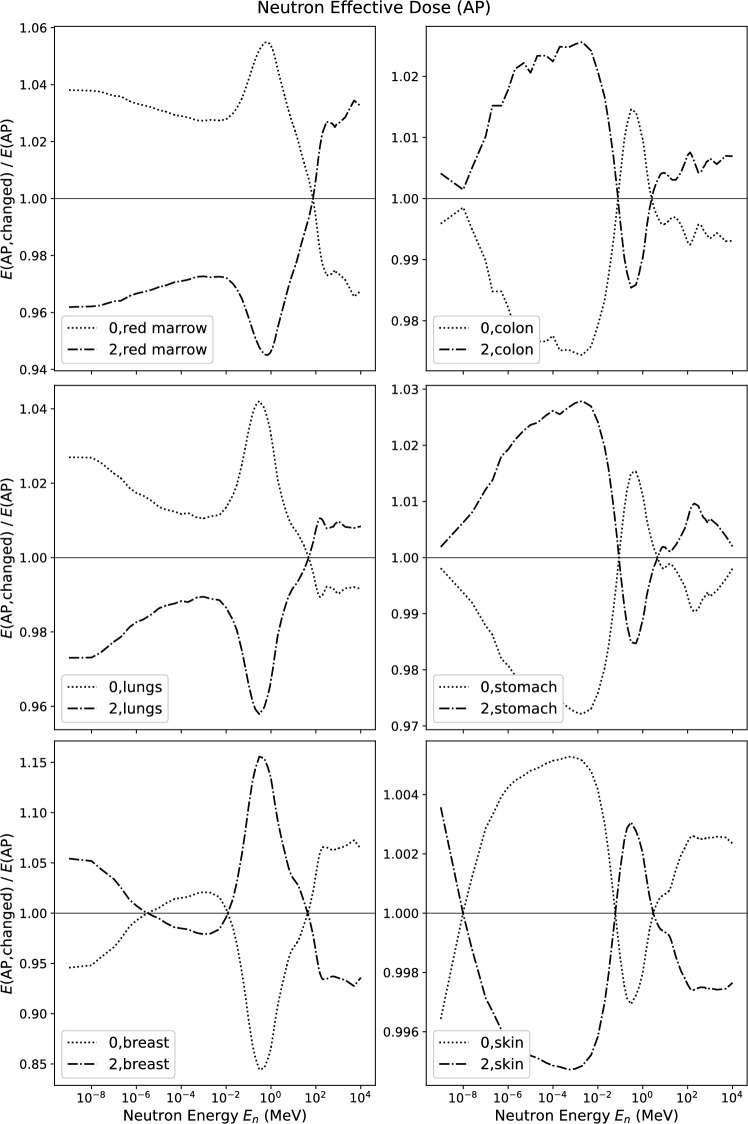
Relative change of effective dose *E* for neutrons in AP incidence when changing the tissue weighting factor indicated in the figure to zero or to its double

**Fig. 7:**
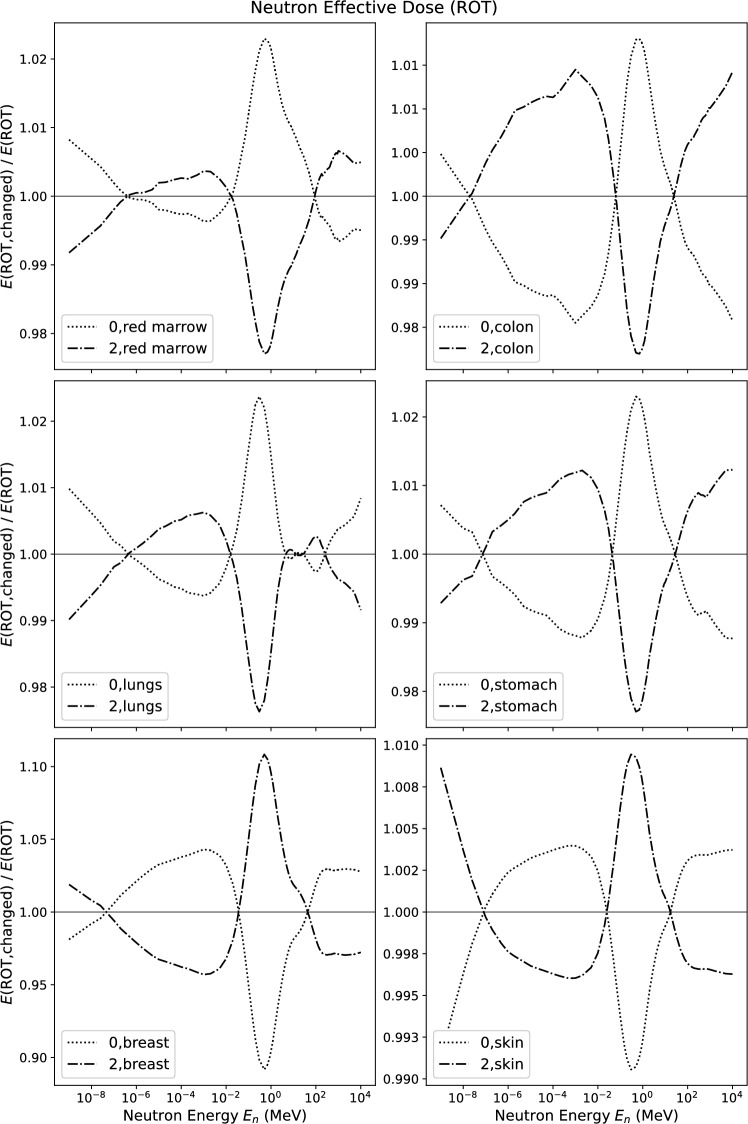
Relative change of effective dose *E* for neutrons in ROT incidence when changing the tissue weighting factor indicated in the figure to zero or to its double

Figures 6 and 7 show the relative changes in effective dose in AP- and ROT-orientation of the radiation field *E*(AP) and *E*(ROT) when the tissue weighting factor $w_{\textrm{T}}$ is either doubled or set to zero. Overall, the relative changes are smaller than is the case for photons, they are mostly lower than 4%. Only the modification of $w_{\textrm{breast}}$ leads to a relative change of effective dose of up to 15% around 1 MeV for AP-orientation. This peak is found with negative sign and lower amplitude for modifications of the other tissue weighting factors due to the compensation mechanism described above. On the contrary to the photon case, skin does not have a significant influence on effective dose at any energy (the apparent peak at 1 MeV in the graphs for skin is more than an order of magnitude lower than the one for the breast).

## Discussion

This work has shown that the sensitivity of effective dose *E* to changes of one of the tissue weighting factors $w_{\textrm{T}}$ by 100% is in the order of a few percent for photon energies higher than 50 keV. For lower photon energies, where effective dose depends predominantly on the contribution of organs close to the body surface (breast, skin), the relative changes amount to up to 60%, with absolute changes in the fluence-to-effective dose conversion coefficient not exceeding 0.15 pSv $\text {cm}^2$. For neutrons, the changes of effective dose *E* are less pronounced at any energy for most tissues, only when the tissue weighting factor for the breast is changed by 100%, a change of *E* by up to 15% can be observed around an energy of 1 MeV.

Realistically it is not expected that in the forthcoming revision of effective dose any weighting factor will change by 100%. The expected changes in *E* after modification of tissue weighting factors will therefore be smaller than the values indicated here.

The operational quantities for external dosimetry introduced in ICRU Report 95 (2020), $H_{\text {p}}$ and $H^*$, are closely related to *E* because they are calculated using the same phantoms and the same weighting factors for radiation and tissue. The concern has been voiced that this close tie would make it necessary to introduce new numerical values for the operational quantities whenever the definition of effective dose would change in the future, and that consequently dosimeters and radiation protection instruments would have to be recalibrated or changed. To this concern, a threefold answer can be formulated:

1. The conversion coefficients in ICRU Report 95 (2020) have been calculated with the numerical reference phantom based on a voxel-geometry and the weighting factors published in ICRP Publication 103 (2007). The next general recommendations of the ICRP will calculate effective dose with mesh-based reference phantoms (ICRP 2020) and likely with changed weighting factors based on the most recent scientific results on radiation effectiveness and sensitivity. Analogously, the conversion coefficients for the operational quantities should be calculated at that moment based on the same models, and on the definitions and methods published in ICRU Report 95.
2. The general recommendations of the ICRP are usually edited for one generation (between the last recommendations in 2007 and the next one, 25 years will have past). This gives sufficient stability for the development of dosimeters and instruments. Actually, developments can be started today with the conversion coefficients from ICRU Report 95, the present work has shown that the expected changes are in the percent range, which is better than the ability of instrument response functions to model the conversion coefficient.
3. Effective dose *E* shows a higher sensitivity to a few tissue weighting factors (breast, skin) at low photon energies. If this should result in significant relative changes of effective dose once the phantoms and weighting factors for the general recommendations of ICRP are frozen, then the absolute changes are still small with respect to effective dose received at other photon energies. Nevertheless, special attention will be required for those groups of persons which are predominantly exposed to low-energy photons.

## Supplementary information

A full set of graphs for the ratios $R_{\mathrm {0,\,T'}}$ and $R_{\mathrm {2,\,T'}}$ for photons and neutrons under all incident directions of the radiation field is found in the supplementary material to the article.

## Supplementary Information

Below is the link to the electronic supplementary material.

Supplementary file 1 (pdf 3400 KB)

## Data Availability

The author declares that the data supporting the findings of this study are available within the paper or its supplementary information files. I submitted the plots for all tissues and all irradiation geometries for photon- and neutron exposure on a as a file " Supplementary Figures" . It comprises 12 pages in A4 format with 15 plots each.

## References

[CR1] Gilvin P, Caresana M, Bottollier-Depois J- F, Chumak V, Clairand I, Eakins J, Behrens R (2022) Evaluation of the Impact of the New ICRU Operational Quantities and Recommendations for their Practical Application (EURADOS Report No. 2022-02). Neuherberg (DE) European Radiation Dosimetry Group

[CR2] ICRP (1991) The 1990 Recommendations of the International Commission on Radiological Protection. ICRP Publication 60. Ann ICRP 21:1–3. 10.1016/0146-6453(91)90065-O 2053748

[CR3] ICRP (1996) Conversion Coefficients for use in Radiological Protection against External Radiation. ICRP Publication 74. Ann ICRP 26:3–4. 10.1016/S0146-6453(97)82921-1 9105539

[CR4] ICRP (2007) The 2007 Recommendations of the International Commission on Radiological Protection. ICRP Publication 103. Annals of the ICRP 37:2–4. 10.1016/j.icrp.2007.10.00 10.1016/j.icrp.2007.10.003 18082557

[CR5] ICRP (2009) Adult Reference Computational Phantoms. ICRP Publication 110. Annals of the ICRP 39:2. 10.1016/j.icrp.2009.07.003 10.1016/j.icrp.2009.09.001 19897132

[CR6] ICRP (2010) Conversion Coefficients for Radiological Protection Quantities for External Radiation Exposures. ICRP Publication 116. Ann ICRP 40:2–5. 10.1016/j.icrp.2011.10.001 10.1016/j.icrp.2011.10.001 22386603

[CR7] ICRP (2020) Adult Mesh-Type Reference Computational Phantoms. ICRP Publication 145. Annals of the ICRP 49(3), 10.1177/0146645319893605 10.1177/0146645319893605 33231095

[CR8] ICRU (1993) ICRU Report 51: Quantities and Units in Radiation Protection Dosimetry (ICRU Report). International Commision on Radiation Units and Measurements, Bethesda, MD, USA. 10.1093/jicru_os26.2.1

[CR9] ICRU (1999) ICRU Report 57: Conversion Coefficients for Use in Radiological Protection against External Radiation ( ICRU Report). International Commision on Radiation Units and Measurements, Bethesda, MD, USA. 10.1093/jicru_os29.2.1

[CR10] ICRU (2020) ICRU Report 95: Operational Quantities for External Radiation Exposure. J ICRU 20:1. 10.1177/1473669120966222

