# Supplementary material for: Sensitivity of effective dose to changes in tissue weighting factors
Source: Radiat Environ Biophys. 2025 Aug 26;64(4):637–47. doi: 10.1007/s00411-025-01143-1 (PMC12701026; doi:10.1007/s00411-025-01143-1)
Supplement: Supplementary file 1 — (pdf 3400 KB) [file 411_2025_1143_MOESM1_ESM.pdf]

Sensitivity of Effective Dose to Changes in Tissue Weighting Factors

T. Otto, CERN, European Organization for Nuclear Research, 1211 Genève 23

Supplementary Figures

The following figures show the ratios $R_{0,T}$, and $R_{2,T}$, (Equation 8 in the paper) for photons and neutrons, for the irradiation geometries AP, PA, LLAT, RLAT, ROT and ISO, and for all 15 tissues identified in ICRP Publication 103, a total of 180 plots grouped in 12 figures.

Figure S1 : $R_{0,T}$, and $R_{2,T}$, for photons and the AP irradiation geometry

Figure S2 : $R_{0,T}$, and $R_{2,T}$, for photons and the PA irradiation geometry

Figure S3 : $R_{0,T}$, and $R_{2,T}$, for photons and the LLAT irradiation geometry

Figure S4 : $R_{0,T}$, and $R_{2,T}$, for photons and the RLAT irradiation geometry

Figure S5 : $R_{0,T}$, and $R_{2,T}$, for photons and the ROT irradiation geometry

Figure S6 : $R_{0,T}$, and $R_{2,T}$, for photons and the ISO irradiation geometry

Figure S7 : $R_{0,T}$, and $R_{2,T}$, for neutrons and the AP irradiation geometry

Figure S8 : $R_{0,T}$, and $R_{2,T}$, for neutrons and the PA irradiation geometry

Figure S9 : $R_{0,T}$, and $R_{2,T}$, for neutrons and the LLAT irradiation geometry

Figure S10 : $R_{0,T}$, and $R_{2,T}$, for neutrons and the RLAT irradiation geometry

Figure S11 : $R_{0,T}$, and $R_{2,T}$, for neutrons and the ROT irradiation geometry

Figure S12 : $R_{0,T}$, and $R_{2,T}$, for neutrons and the ISO irradiation geometry

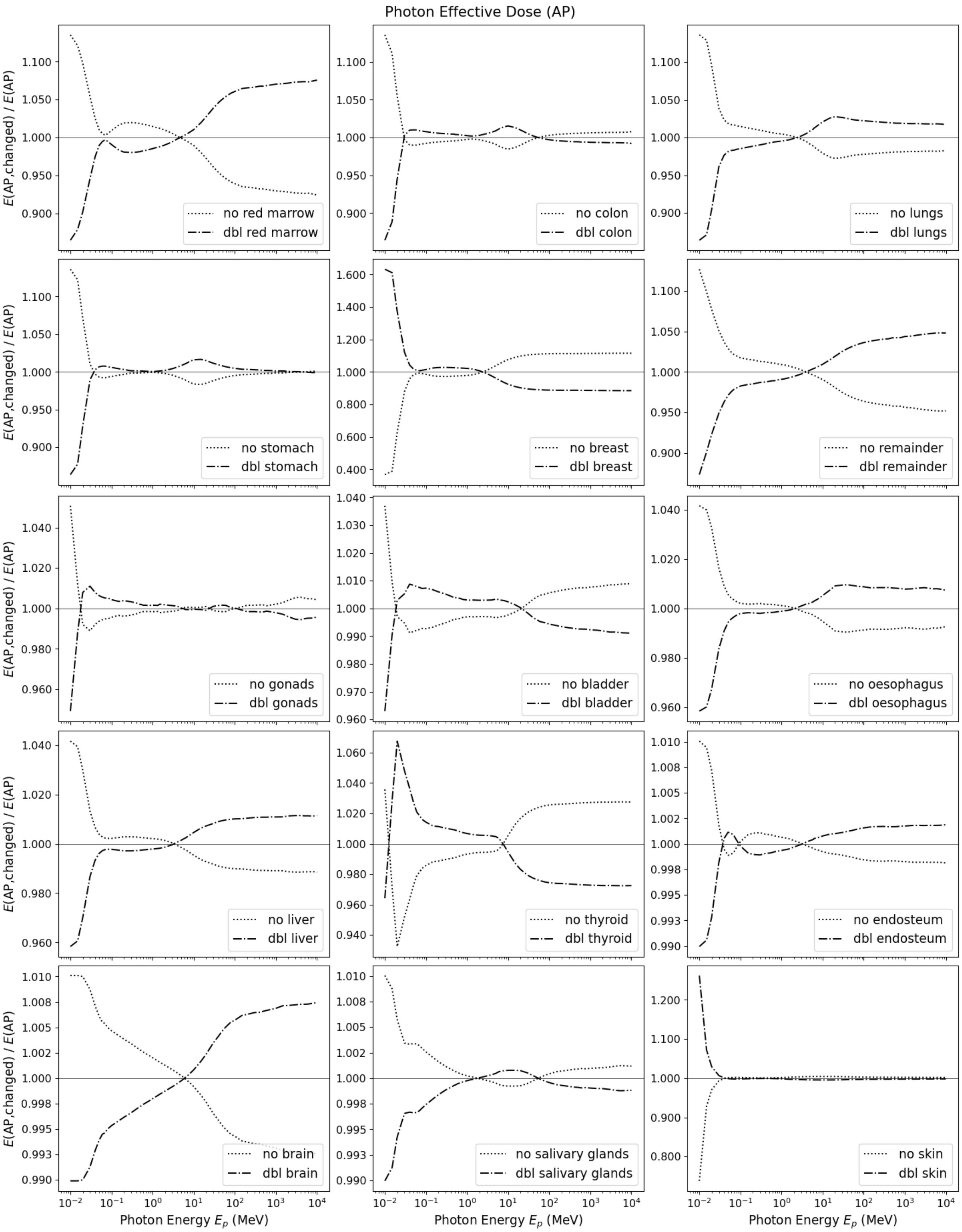

Photon Effective Dose (AP)
E(AP,changed) / E(AP)
no red marrow
dbl red marrow
no colon
dbl colon
no lungs
dbl lungs
no stomach
dbl stomach
no breast
dbl breast
no remainder
dbl remainder
no gonads
dbl gonads
no bladder
dbl bladder
no oesophagus
dbl oesophagus
no liver
dbl liver
no thyroid
dbl thyroid
no endosteum
dbl endosteum
no brain
dbl brain
no salivary glands
dbl salivary glands
no skin
dbl skin
Photon Energy E_p (MeV)

Figure S1

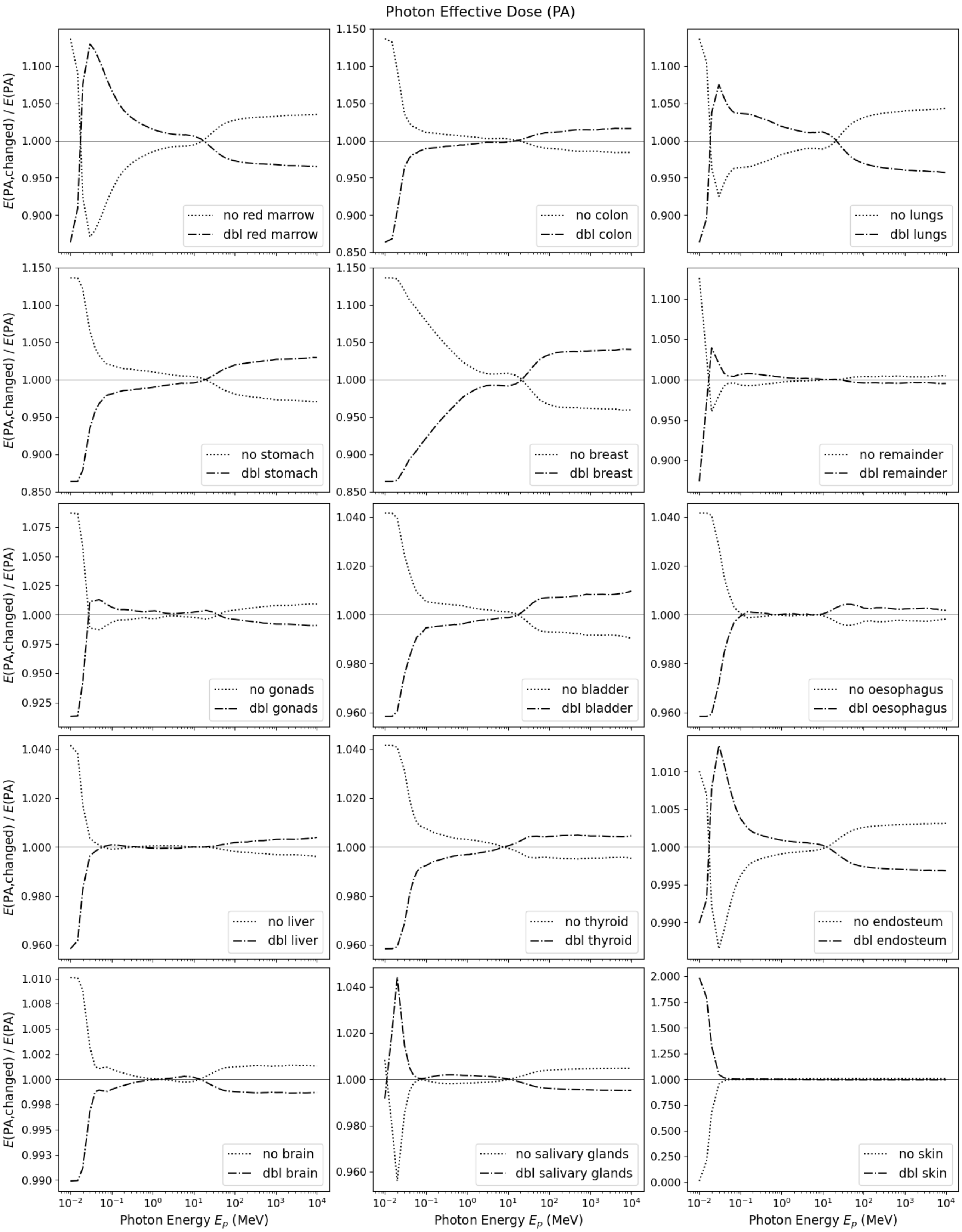
Photon Effective Dose (PA)
E(PA,changed) / E(PA)
Photon Energy E_p (MeV)
no red marrow
dbl red marrow
no colon
dbl colon
no lungs
dbl lungs
no stomach
dbl stomach
no breast
dbl breast
no remainder
dbl remainder
no gonads
dbl gonads
no bladder
dbl bladder
no oesophagus
dbl oesophagus
no liver
dbl liver
no thyroid
dbl thyroid
no endosteum
dbl endosteum
no brain
dbl brain
no salivary glands
dbl salivary glands
no skin
dbl skin

Figure S2

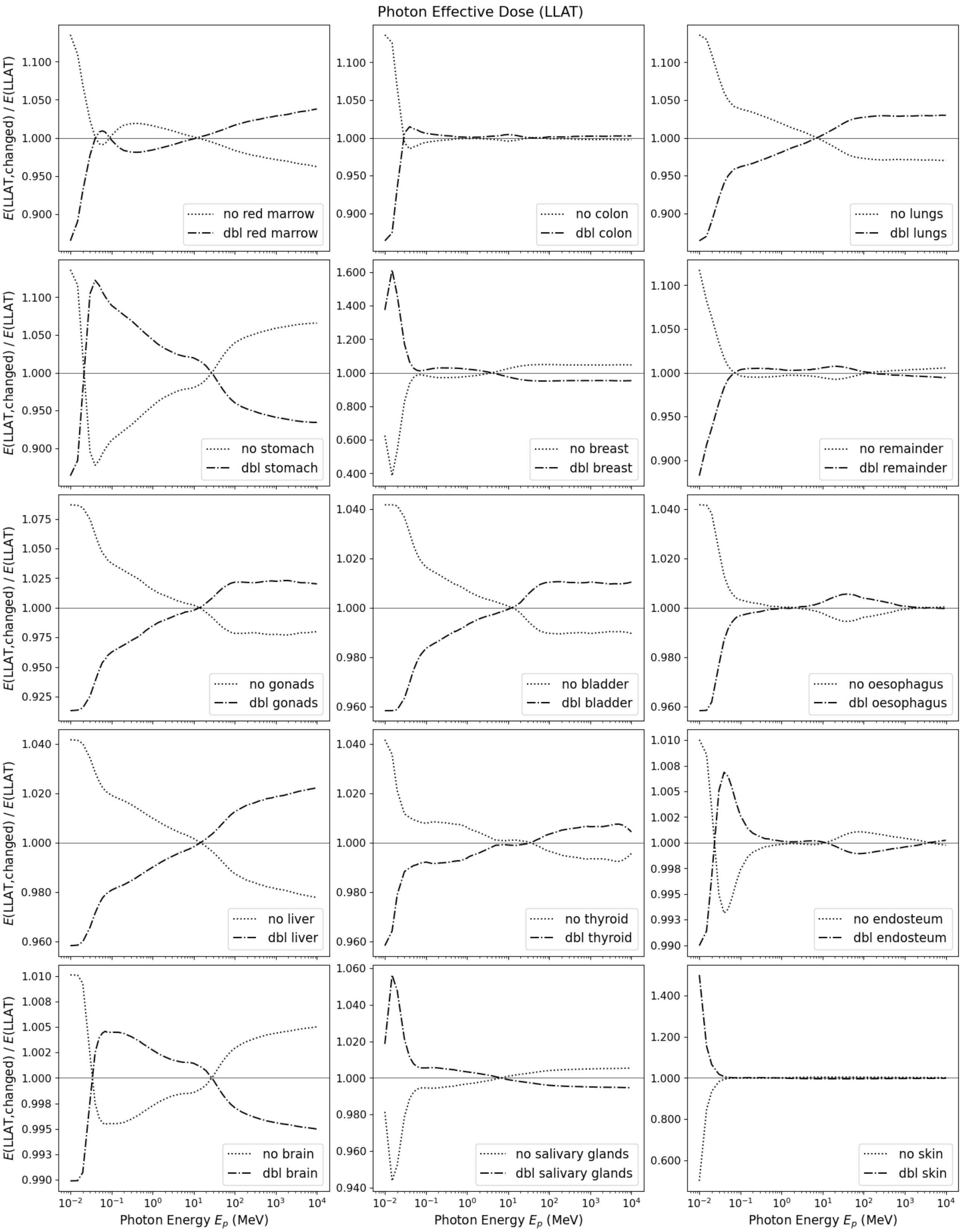

Photon Effective Dose (LLAT)
E(LLAT,changed) / E(LLAT)
Photon Energy E_p (MeV)
no red marrow
dbl red marrow
no colon
dbl colon
no lungs
dbl lungs
no stomach
dbl stomach
no breast
dbl breast
no remainder
dbl remainder
no gonads
dbl gonads
no bladder
dbl bladder
no oesophagus
dbl oesophagus
no liver
dbl liver
no thyroid
dbl thyroid
no endosteum
dbl endosteum
no brain
dbl brain
no salivary glands
dbl salivary glands
no skin
dbl skin

Figure S3

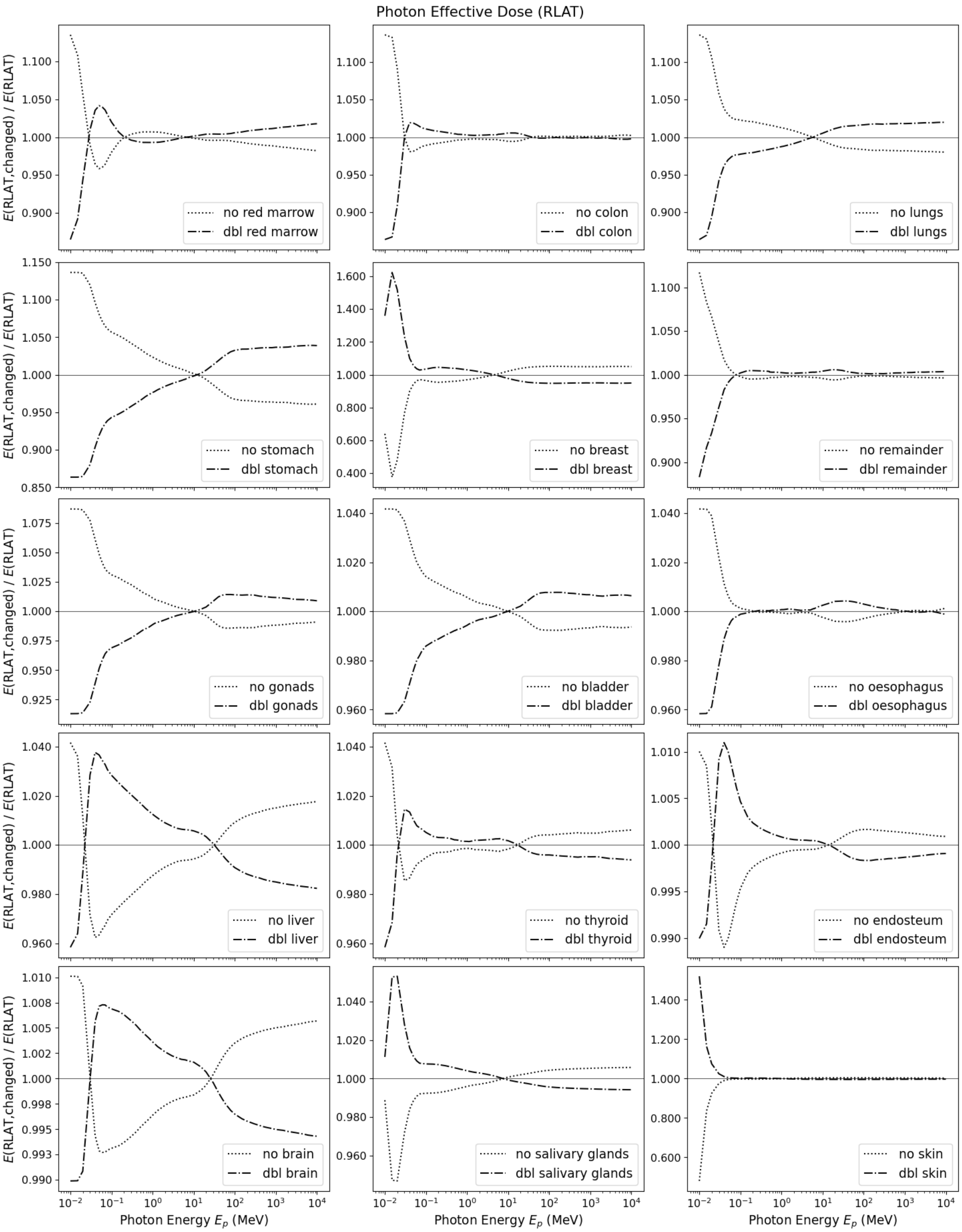
Photon Effective Dose (RLAT)
E(RLAT,changed) / E(RLAT)
no red marrow
dbl red marrow
no colon
dbl colon
no lungs
dbl lungs
no stomach
dbl stomach
no breast
dbl breast
no remainder
dbl remainder
no gonads
dbl gonads
no bladder
dbl bladder
no oesophagus
dbl oesophagus
no liver
dbl liver
no thyroid
dbl thyroid
no endosteum
dbl endosteum
no brain
dbl brain
no salivary glands
dbl salivary glands
no skin
dbl skin
Photon Energy E_p (MeV)

Figure S4

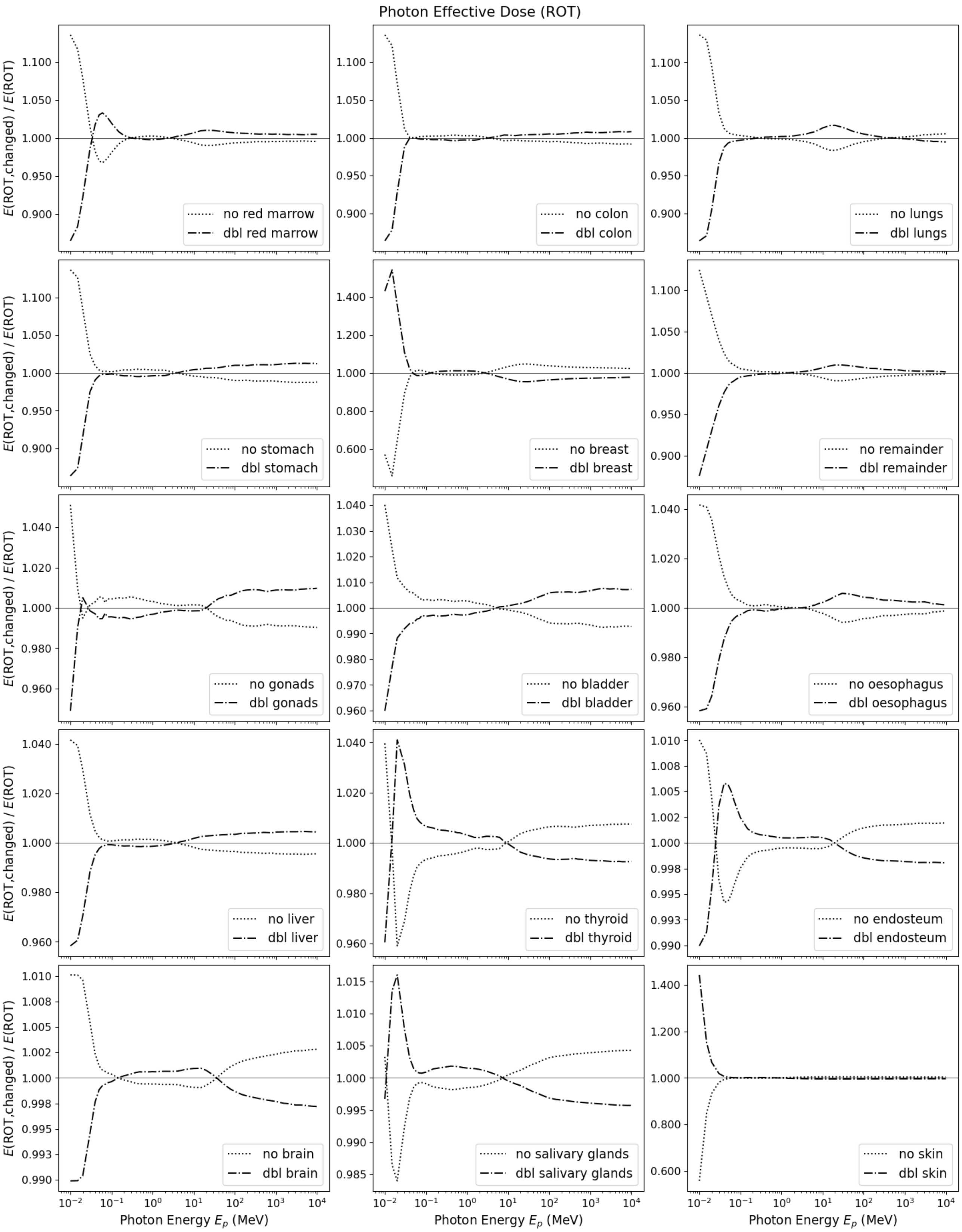
Photon Effective Dose (ROT)
E(ROT,changed) / E(ROT)
no red marrow
dbl red marrow
no colon
dbl colon
no lungs
dbl lungs
no stomach
dbl stomach
no breast
dbl breast
no remainder
dbl remainder
no gonads
dbl gonads
no bladder
dbl bladder
no oesophagus
dbl oesophagus
no liver
dbl liver
no thyroid
dbl thyroid
no endosteum
dbl endosteum
no brain
dbl brain
no salivary glands
dbl salivary glands
no skin
dbl skin
Photon Energy E_p (MeV)

Figure S5

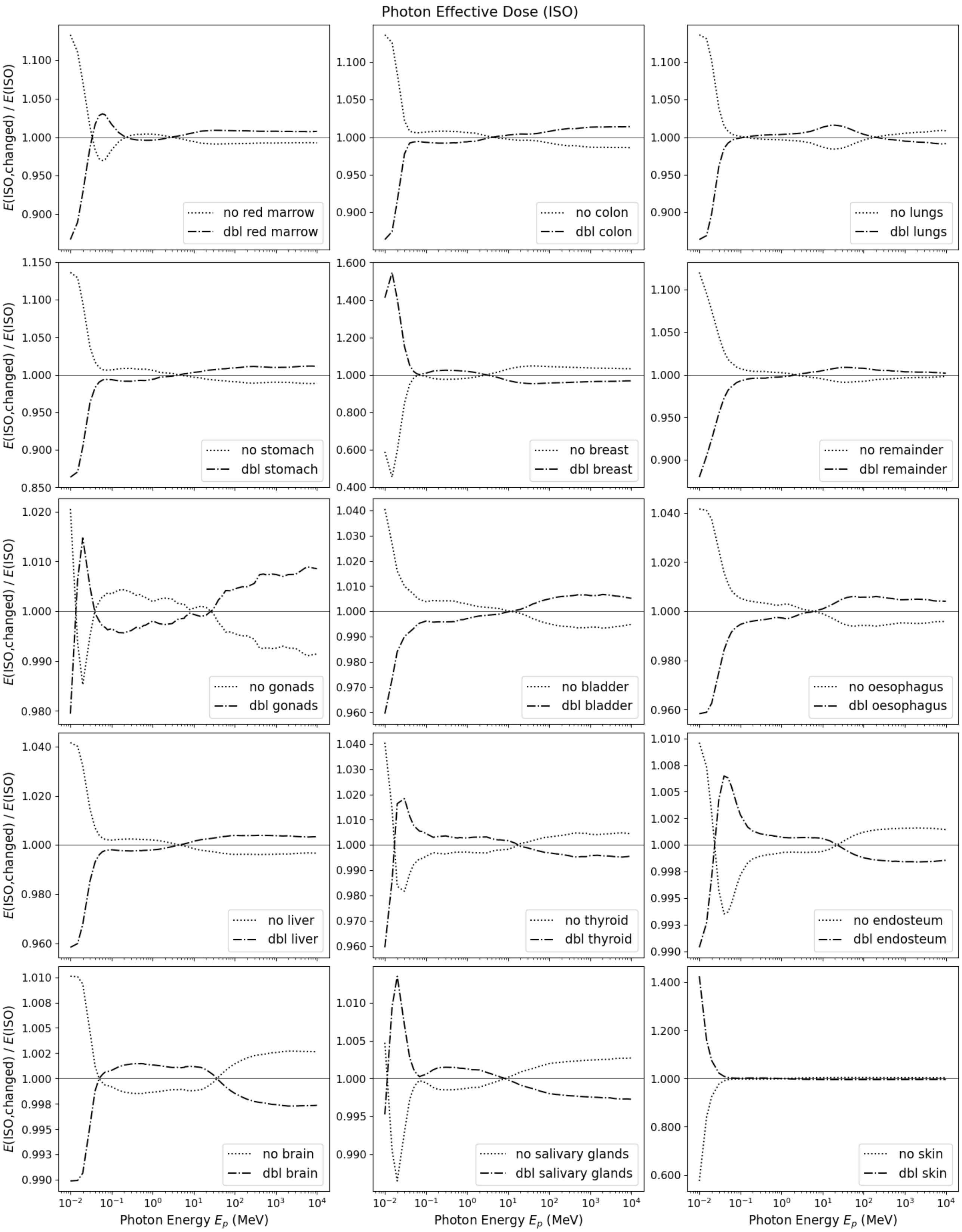

Photon Effective Dose (ISO)
E(ISO,changed) / E(ISO)
Photon Energy E_p (MeV)
no red marrow
dbl red marrow
no colon
dbl colon
no lungs
dbl lungs
no stomach
dbl stomach
no breast
dbl breast
no remainder
dbl remainder
no gonads
dbl gonads
no bladder
dbl bladder
no oesophagus
dbl oesophagus
no liver
dbl liver
no thyroid
dbl thyroid
no endosteum
dbl endosteum
no brain
dbl brain
no salivary glands
dbl salivary glands
no skin
dbl skin

Figure S6

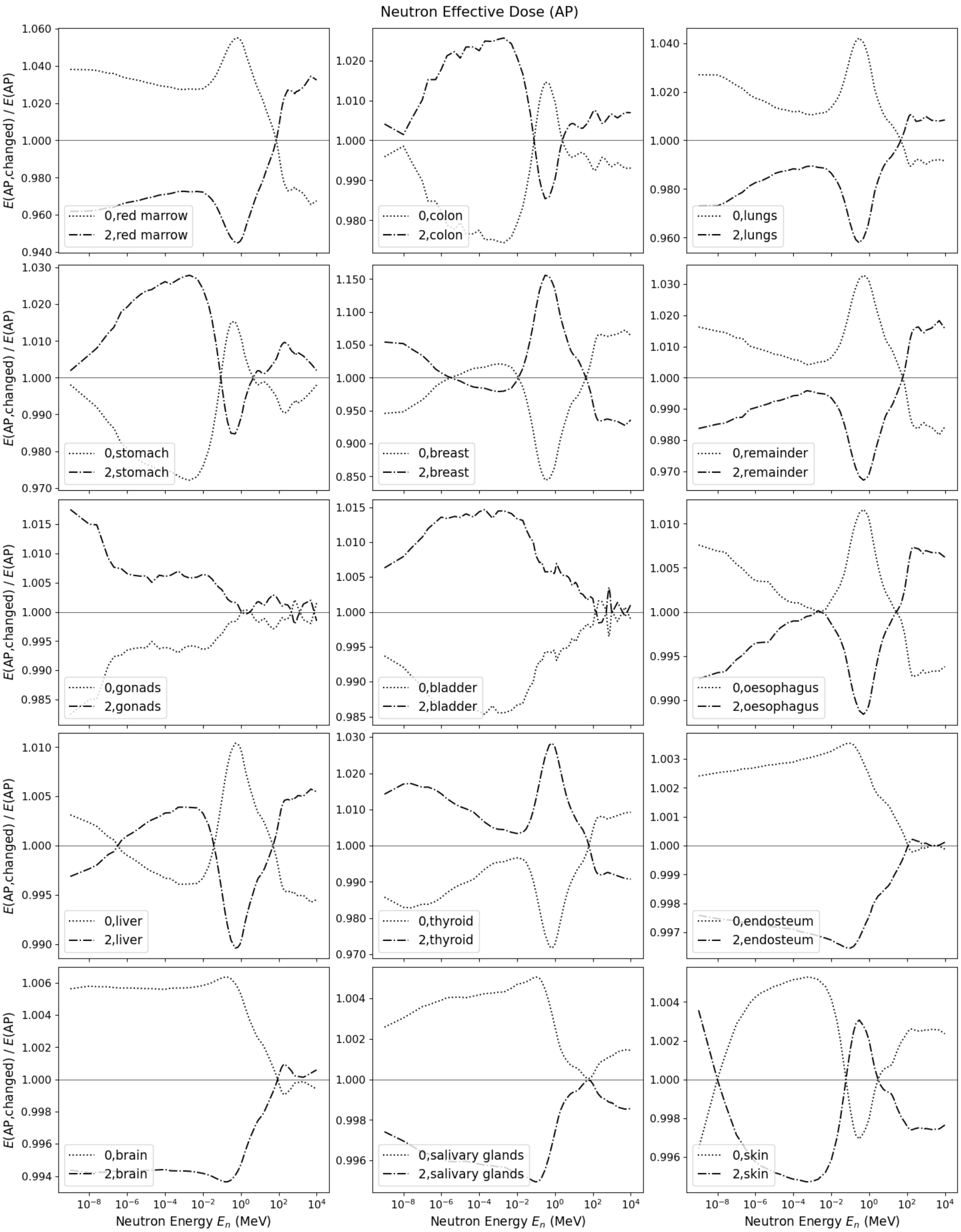
Neutron Effective Dose (AP)
E(AP,changed) / E(AP)
Neutron Energy E_n (MeV)
0,red marrow
2,red marrow
0,colon
2,colon
0,lungs
2,lungs
0,stomach
2,stomach
0,breast
2,breast
0,remainder
2,remainder
0,gonads
2,gonads
0,bladder
2,bladder
0,oesophagus
2,oesophagus
0,liver
2,liver
0,thyroid
2,thyroid
0,endosteum
2,endosteum
0,brain
2,brain
0,salivary glands
2,salivary glands
0,skin
2,skin

Figure S7

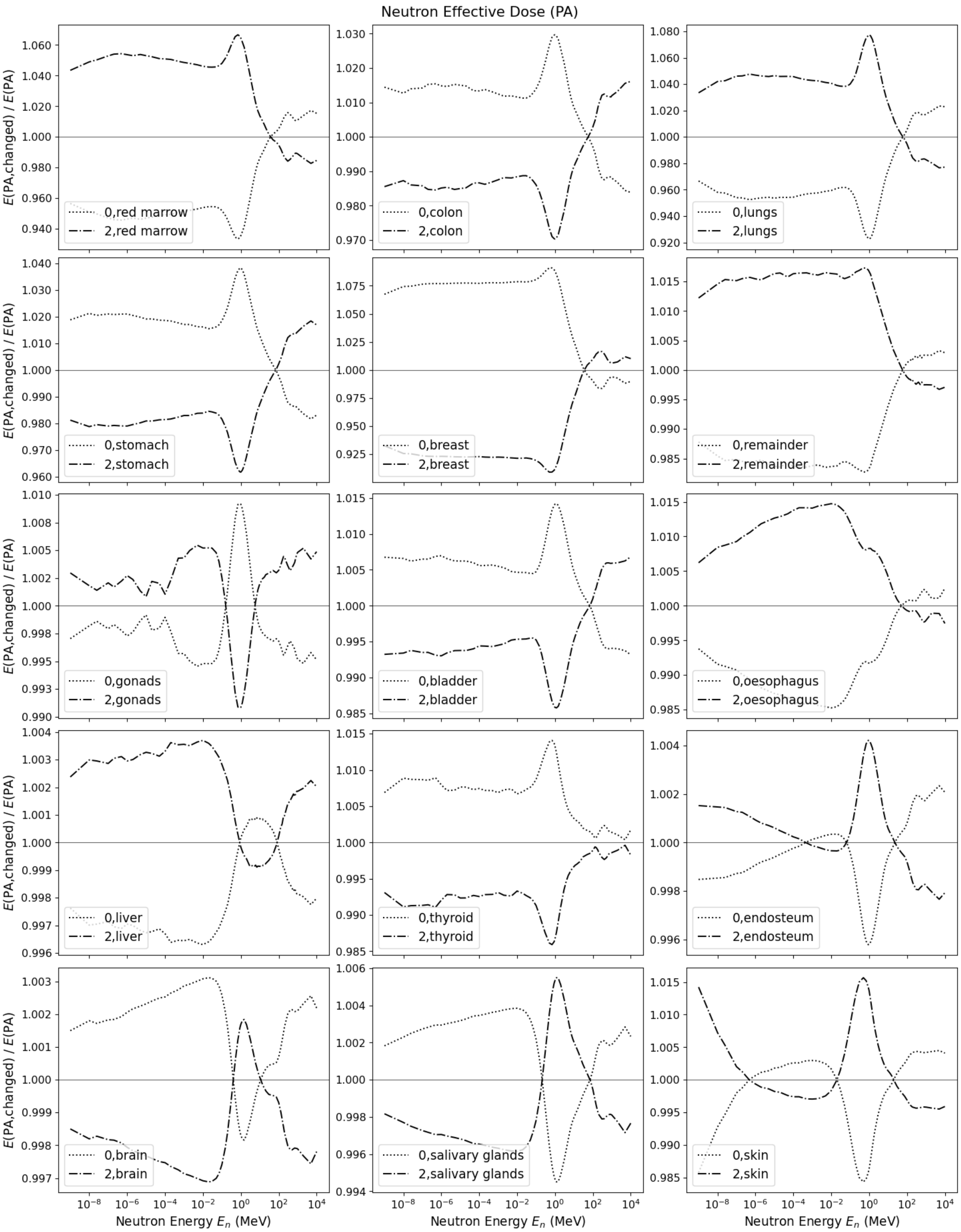
Neutron Effective Dose (PA)
E(PA,changed) / E(PA)
0,red marrow
2,red marrow
0,colon
2,colon
0,lungs
2,lungs
0,stomach
2,stomach
0,breast
2,breast
0,remainder
2,remainder
0,gonads
2,gonads
0,bladder
2,bladder
0,oesophagus
2,oesophagus
0,liver
2,liver
0,thyroid
2,thyroid
0,endosteum
2,endosteum
0,brain
2,brain
0,salivary glands
2,salivary glands
0,skin
2,skin
Neutron Energy E_n (MeV)

Figure S8

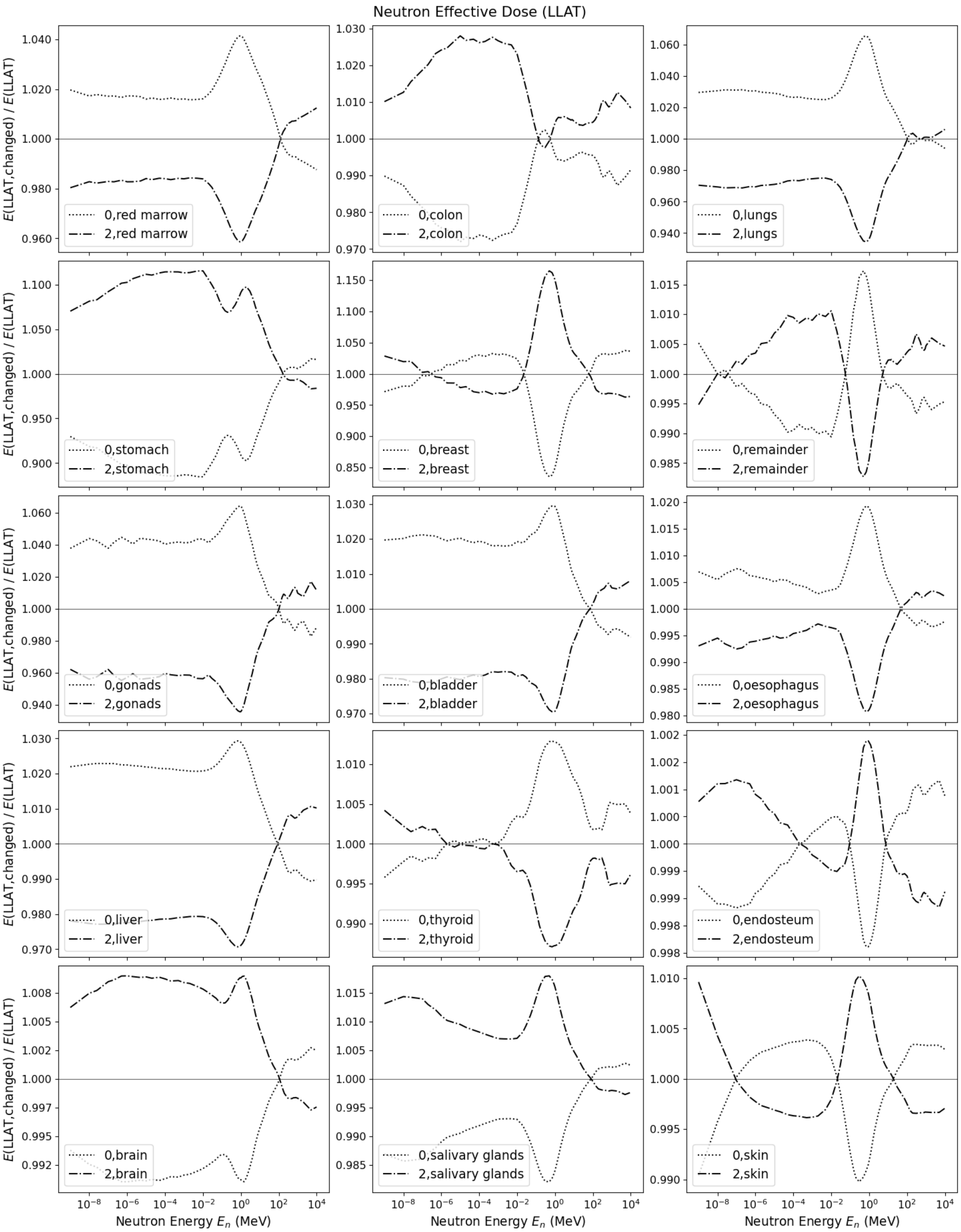

Neutron Effective Dose (LLAT)
E(LLAT,changed) / E(LLAT)
Neutron Energy E_n (MeV)
0,red marrow
2,red marrow
0,colon
2,colon
0,lungs
2,lungs
0,stomach
2,stomach
0,breast
2,breast
0,remainder
2,remainder
0,gonads
2,gonads
0,bladder
2,bladder
0,oesophagus
2,oesophagus
0,liver
2,liver
0,thyroid
2,thyroid
0,endosteum
2,endosteum
0,brain
2,brain
0,salivary glands
2,salivary glands
0,skin
2,skin

Figure S9

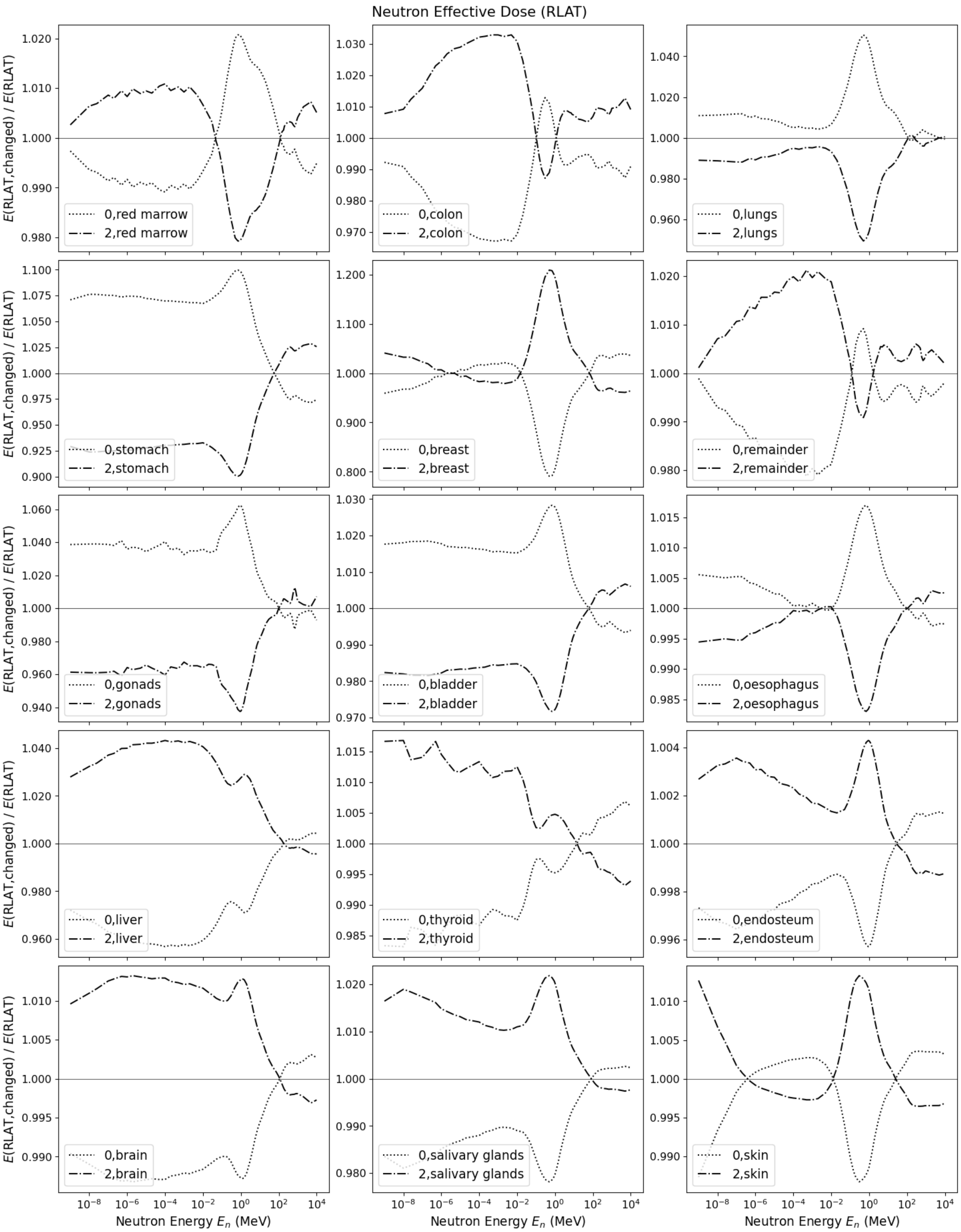

Neutron Effective Dose (RLAT)
E(RLAT,changed) / E(RLAT)
0,red marrow
2,red marrow
0,colon
2,colon
0,lungs
2,lungs
0,stomach
2,stomach
0,breast
2,breast
0,remainder
2,remainder
0,gonads
2,gonads
0,bladder
2,bladder
0,oesophagus
2,oesophagus
0,liver
2,liver
0,thyroid
2,thyroid
0,endosteum
2,endosteum
0,brain
2,brain
0,salivary glands
2,salivary glands
0,skin
2,skin
Neutron Energy E_n (MeV)

Figure S10

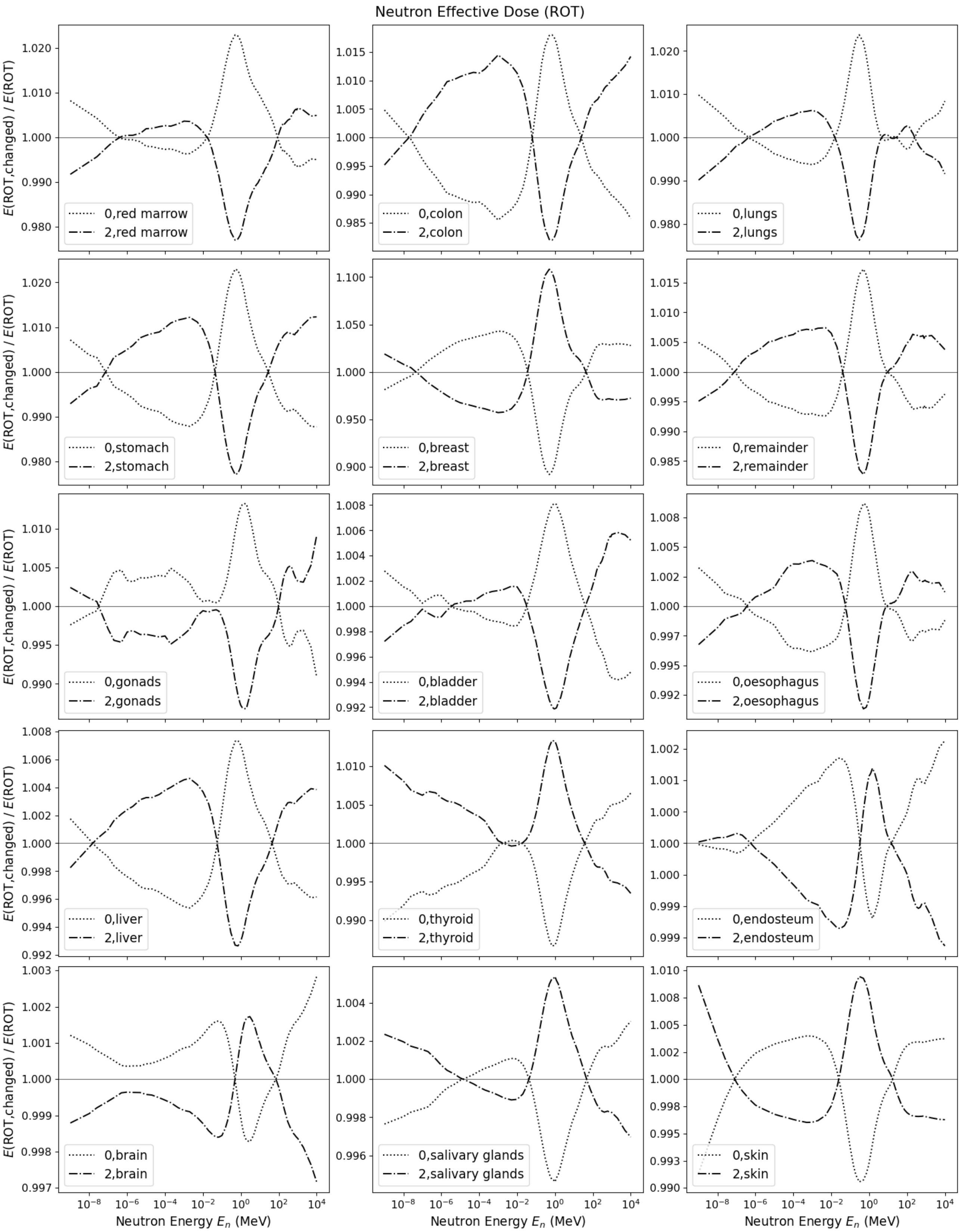
Neutron Effective Dose (ROT)
E(ROT,changed) / E(ROT)
Neutron Energy E_n (MeV)
0,red marrow
2,red marrow
0,colon
2,colon
0,lungs
2,lungs
0,stomach
2,stomach
0,breast
2,breast
0,remainder
2,remainder
0,gonads
2,gonads
0,bladder
2,bladder
0,oesophagus
2,oesophagus
0,liver
2,liver
0,thyroid
2,thyroid
0,endosteum
2,endosteum
0,brain
2,brain
0,salivary glands
2,salivary glands
0,skin
2,skin

Figure S11

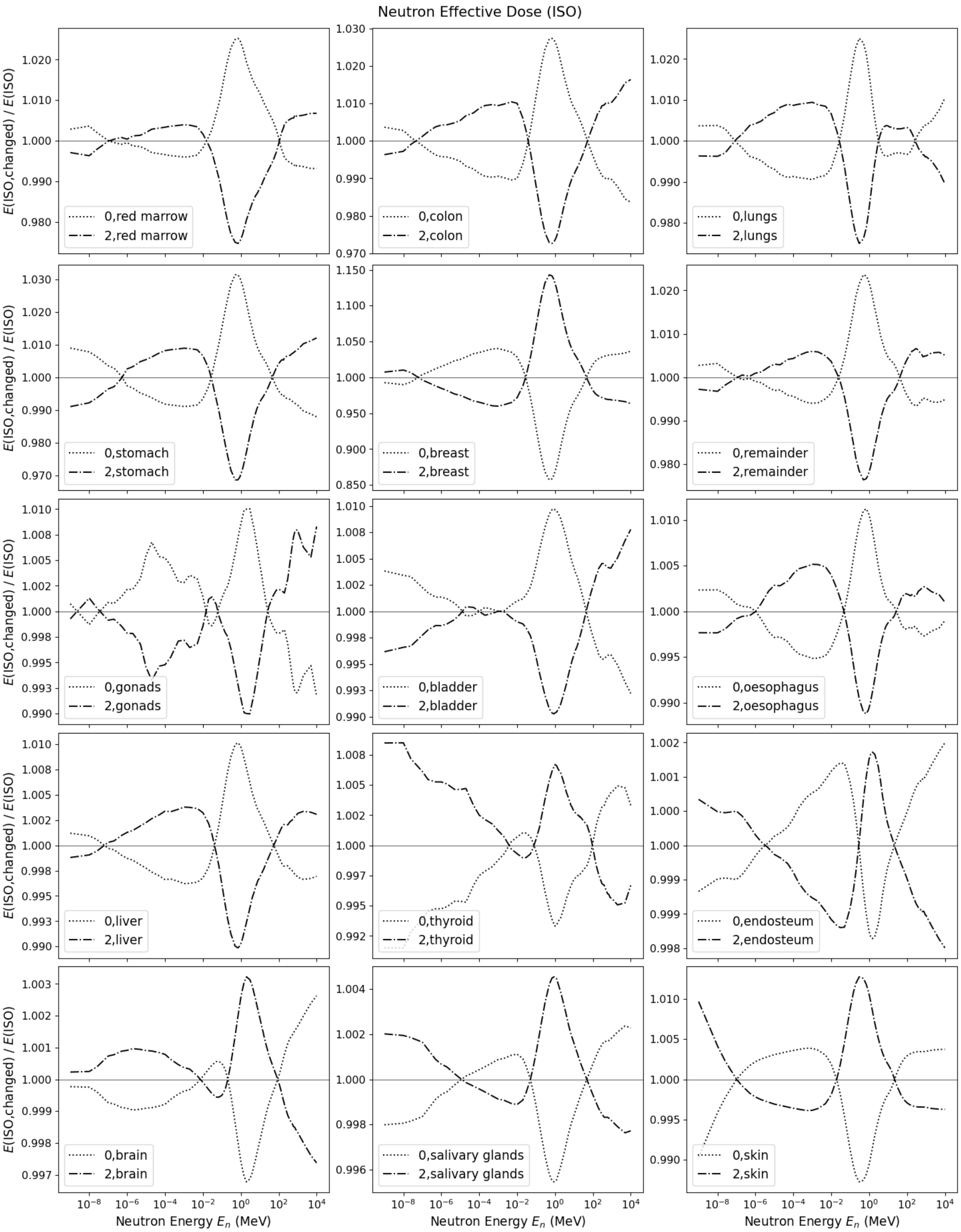
Neutron Effective Dose (ISO)
E(ISO,changed) / E(ISO)
Neutron Energy E_n (MeV)
0,red marrow
2,red marrow
0,colon
2,colon
0,lungs
2,lungs
0,stomach
2,stomach
0,breast
2,breast
0,remainder
2,remainder
0,gonads
2,gonads
0,bladder
2,bladder
0,oesophagus
2,oesophagus
0,liver
2,liver
0,thyroid
2,thyroid
0,endosteum
2,endosteum
0,brain
2,brain
0,salivary glands
2,salivary glands
0,skin
2,skin

Figure S12
